# Immunological Reactivity Using Monoclonal and Polyclonal Antibodies of Autoimmune Thyroid Target Sites with Dietary Proteins

**DOI:** 10.1155/2017/4354723

**Published:** 2017-08-15

**Authors:** Datis Kharrazian, Martha Herbert, Aristo Vojdani

**Affiliations:** ^1^Harvard Medical School, Boston, MA, USA; ^2^TRANSCEND Research, Department of Neurology, Massachusetts General Hospital, Charlestown, MA 02129, USA; ^3^Department of Preventive Medicine, Loma Linda University School of Medicine, Loma Linda, CA, USA; ^4^Immunosciences Laboratory, Inc., Los Angeles, CA, USA

## Abstract

Many hypothyroid and autoimmune thyroid patients experience reactions with specific foods. Additionally, food interactions may play a role in a subset of individuals who have difficulty finding a suitable thyroid hormone dosage. Our study was designed to investigate the potential role of dietary protein immune reactivity with thyroid hormones and thyroid axis target sites. We identified immune reactivity between dietary proteins and target sites on the thyroid axis that includes thyroid hormones, thyroid receptors, enzymes, and transport proteins. We also measured immune reactivity of either target specific monoclonal or polyclonal antibodies for thyroid-stimulating hormone (TSH) receptor, 5′deiodinase, thyroid peroxidase, thyroglobulin, thyroxine-binding globulin, thyroxine, and triiodothyronine against 204 purified dietary proteins commonly consumed in cooked and raw forms. Dietary protein determinants included unmodified (raw) and modified (cooked and roasted) foods, herbs, spices, food gums, brewed beverages, and additives. There were no dietary protein immune reactions with TSH receptor, thyroid peroxidase, and thyroxine-binding globulin. However, specific antigen-antibody immune reactivity was identified with several purified food proteins with triiodothyronine, thyroxine, thyroglobulin, and 5′deiodinase. Laboratory analysis of immunological cross-reactivity between thyroid target sites and dietary proteins is the initial step necessary in determining whether dietary proteins may play a potential immunoreactive role in autoimmune thyroid disease.

## 1. Introduction

Immunologic cross-reactivity occurs when adaptive immune response against one antigen also occurs to another antigen with amino acid structural similarity. Immunological cross-reactivity was first identified in 1942 when it was found that individuals sensitized to pollen allergens developed immune reactivity to specific fruits [1]. Further study found that cross-reactivity with pollen could also occur to human tissue target proteins [2].

Exposure to antigens that share amino acid sequence homology with self-tissue proteins in susceptible hosts has been theorized as a trigger for tissue-specific autoimmune disease [3, 4].

Various antigens have been shown to specifically cross-react with thyroid tissue and trigger thyroid autoimmunity. Additionally, heat shock protein 60 (Hsp60), a mitochondrial chaperonin involved in stress responses, diabetes, and immunological disorder, has structural similarity to thyroglobulin and thyroid peroxidase molecules. Enzyme-linked immunosorbent assay (ELISA) evaluations have also shown immunological cross-reactivity to play a role in Hashimoto's thyroiditis [5].

Numerous gastrointestinal pathogens have demonstrated molecular mimicry with thyroid tissue. For example, human monoclonal thyroid-stimulating hormone receptor (TSH-r) has been shown to cross-react with* Yersinia enterocolitica (Y. enterocolitica)*, thereby providing a mechanistic framework for molecular mimicry in Graves' disease, where* Y. enterocolitica* antibody production promotes cross-reactive pathogenic response to TSH receptor [6]. Researchers have discovered that the outer membrane of the porin F protein of* Y. enterocolitica* shares cross-immunogenicity with a leucine-rich domain of TSH receptor and plays a role in inducing autoimmunity to TSH receptor through molecular mimicry [7].

Immune cross-reactivity between* Helicobacter pylori (H. pylori)* and autoimmune thyroid disease has been suspected due to correlations between early onset* H. pylori infections* and Hashimoto's hypothyroidism and Graves' disease [8, 9]. Cross-reactivity with* Candida albicans* and thyroid antigens has been identified and associated with the development of autoimmune thyroid disease [10]. The protozoa* Toxoplasma gondii* has also been reported to potentially induce autoimmune thyroid cross-reactivity through molecular mimicry mechanisms [11].

In addition to gastrointestinal pathogens,* Clostridium botulinum* neurotoxin A (Btx) has been reported to induce serum elevations of TSH. Researchers found that Btx and thyroid autoantigens share amino acid sequence homology and may play a role in cross-reactive complication of autoimmune thyroid disease [12].* Borrelia burgdorferi* has been shown to have protein homology with TSH receptor and therefore plays a role as an antigenic trigger for autoimmune thyroid disease [13].* Coxsackie* virus antibodies have also demonstrated cross-reactivity with the thyroid and have been reported to be a contributing factor to the pathogenesis of autoimmune thyroid disorder [14].

The list of pathogenic organisms, such as viral, bacterial, fungal, spirochete, and protozoa antibodies, that may contribute to tissue-specific thyroid autoimmunity via antibody amino acid sequence homology is a growing field of study. However, little research has been done with food protein antibodies and their potential role in thyroid specific cross-reactivity. Published research has looked at gluten and thyroid autoimmunity, but the immunological reactive role that other dietary proteins may play with thyroid function has been a limited area of research [15, 16].

In this study, we evaluated the potential for food cross-reactivity with the thyroid axis by evaluating immune reactivity between purified dietary proteins and thyroid target specific antibodies. We measured immune reactivity of either target specific monoclonal or polyclonal antibodies for TSH receptor, 5′deiodinase, thyroid peroxidase, thyroglobulin, thyroxine-binding globulin, thyroxine, and triiodothyronine against 204 purified dietary proteins that are commonly consumed in raw or cooked forms. Food determinants included unmodified (raw) and modified (cooked and roasted) food proteins, herbs, spices, food gums, brewed beverages, and additives. We included raw versus modified food antigens since in our earlier study we showed that some individuals may react to raw food antigens but not heat-modified ones, while, conversely, others may react to heat-modified but not raw food antigens [17].

Specific target proteins along the thyroid axis associated with autoimmune thyroid disease were evaluated in our study including thyroid-stimulating hormone receptor (TSH-R), 5′deiodinase, thyroid peroxidase, thyroglobulin, thyroxine-binding globulin, thyroxine, and triiodothyronine. TSH-R antibody reactions are the hallmark of Graves' disease. These antibody reactions promote autoimmune reactivity against the target protein, but homosteric binding to these receptors can promote increased production of thyroid hormones, leading to hyperthyroidism [18]. Thyroid peroxidase (TPO) is the key thyroid enzyme necessary for the synthesis of thyroid hormones and is the target for Hashimoto's autoimmunity. TPO antibodies are associated with destruction of the thyroid and thyroiditis [19]. Thyroglobulin (Tg) is a dimeric protein synthesized in the thyroid and used for production of thyroid hormones. Its level is elevated with thyroid tissue breakdown, such as with thyroiditis and differentiated thyroid cancer [20]. Tg is a common target for thyroid autoimmunity [21]. Thyroxine-binding globulin (TBG) is the transport protein for thyroid hormones in circulation and has high affinity for thyroxine and triiodothyronine [22]. Levels in the body can change due to metabolic disease, pregnancy, oral contraceptive use, and hormone replacement therapy [23]. Type II iodothyronine deiodinase (DIO_2_) is the enzyme that converts prohormone thyroxine by outer ring deiodination to bioactive triiodothyronine [24]. Thyroxine (T_4_) is a prohormone synthesized by the thyroid gland and composed of four iodine molecules attached to thyroglobulin. Triiodothyronine (T_3_) is bioactive thyroid hormone that is responsible for the key physiological mechanisms of thyroid target tissue function [25].

The identification of immunological food protein cross-reactivity in the laboratory with thyroid axis target sites is the first step in understanding whether dietary proteins may potentially play an immunological reactive role in autoimmune thyroid disease. In this study we attempt to take the initial first step by determining any potential patterns of immunological cross-reactivity with a diverse list of food proteins an specific thyroid axis autoimmune target sites.

## 2. Materials and Methods

### 2.1. Polyclonal and Monoclonal Antibodies

Affinity-purified rabbit polyclonal thyroid-stimulating hormone receptor antibody, affinity-purified goat polyclonal thyroxine 5-deiodinase (DIO_2_) antibody, monoclonal antibody to thyroid peroxidase, monoclonal antibody against thyroglobulin, mouse monoclonal thyroxine antibody, mouse monoclonal antibody to triiodothyronine, and monoclonal antibody to thyroxine-binding globulin were purchased from MyBioSource, Inc. (San Diego, CA, USA).

### 2.2. Preparation of Dietary Antigens

Food antigens were prepared from products purchased from the supermarket in both raw, roasted, or cooked form. For that preparation, 10 g of food product was put in a food processor using 0.1 M of phosphate buffer saline (PBS) at pH 7.4. The mixer was turned on and off for 1 hour and then kept on the stirrer overnight at 4°C. After centrifugation at 20,000*g* for 15 minutes, the top layer, which contained oil bodies, was discarded. The liquid phase was removed and dialyzed against 0.01 M of PBS using dialysis bags, with a cutoff of 6,000 kDa. Dialysis was repeated three times to ensure all small molecules were removed. After dialysis, all samples were filtered through a 0.2 micron filter to remove any debris. Protein concentrations were measured using a kit provided by Bio-Rad (Hercules, CA, USA). Different peptides were purchased from Bio-Synthesis (Lewisville, TX, USA). Lectin and agglutinins were purchased from Sigma Aldrich (St. Louis, MO, USA).

### 2.3. Preparation of Dietary Oleosin Antigens

To purify the oleosin from peanuts, corn, safflower, sunflower, and soybean, the foods were prepared according to the method described by Vojdani [26]. A total of 100 mL of chloroform/methanol (2/1, v/v) was then added and blended for 2 minutes using a food processor. The mixture was put in a 50 mL tube and centrifuged at 14,000 RPM for 5 minutes. The liquid in the upper phase was filtered through two layers of filter paper. The resultant filtrate was collected in multiple glass bottles and dried under a stream of air, with strong continuous agitation. The chloroform/methanol extraction step was repeated twice. A total of 20 mL of diethyl ether was then added, and the white, solid material stuck on the surface of the glass bottles was detached and resuspended in diethyl ether. At this point, 10 mL of water was added to each bottle, which was centrifuged at 20,000*g* for 5 minutes. The upper diethyl ether layer that contained lipids was removed, and the white, solid, interface material containing the oleosins was collected and transferred to microtubes with a minimum volume of water and diethyl ether. The microtubes were centrifuged at 20,000*g* for 5 minutes. The interfacial material was exposed to a stream of nitrogen to evaporate the remaining diethyl ether. One mL of chloroform/ethanol (95/5, v/v) was added to the interfacial material in each tube. The contents of each tube were quickly vortexed and transferred to a glass flask. To separate any protein contaminants from the oleosins, 10 mL of chloroform/methanol (95/5, v/v) was added, and the mixture was filtered through filter paper that was previously rinsed with chloroform/methanol. The filtrate was collected in a flask and dried under a stream of nitrogen. The dried oleosins were dissolved in chloroform/methanol and applied to a Sephadex LH-60 column (Bio-Rad, Hercules, CA, USA) using chloroform/methanol as the solvent. The collected fractions of oleosins were checked by sodium dodecyl sulfate (SDS)-gel electrophoresis.

### 2.4. Preparation of Dietary Gum Antigens

Mastic gum, carrageenan, xanthan gum, guar gum, gum tragacanth, locust bean gum, and *β*-glucan were purchased from Sigma Aldrich (Saint Louis, MO, USA). Extracts from these items were prepared according to the procedures described by Vojdani [27]. Ten grams of each gum was extracted in 500 mL of buffer pH 4.6 by mixing them for 8 hours at 25°C on a magnetic stirrer. The solution was centrifuged at 20,000*g*, and supernatant was removed and concentrated by a factor of 10 using an Amicon filter. The protein concentration was measured using a kit provided by Bio-Rad (Hercules, CA, USA). All extracts were aliquoted and stored frozen at −20°C until used. Different gum extracts were dissolved in 0.1 M PBS. These antigens were diluted 1 : 50 in 0.1 M carbonate buffer pH 9.2 and 100 *μ*L of each gum antigen.

### 2.5. Enzyme-Linked Immunosorbent Assay (ELISA) for Demonstration of Immune Reactivity

Food antigens and peptides were dissolved in PBS or methanol at a concentration of 1.0 mg/mL and then diluted 1 : 100 in 0.1 M carbonate-bicarbonate buffer at a pH of 9.5 and 100 *μ*L was added to each well of the polystyrene flat-bottom ELISA plate. Plates were incubated overnight at 4°C and then washed three times with 200 *μ*L Tris-buffered Saline (TBS) containing 0.05% Tween 20 at a pH of 7.4. The nonspecific binding of immunoglobulins was prevented by adding a mixture of 2% bovine serum albumin (BSA) into the TBS and then incubating overnight at 4°C. Plates were washed as described above, and then serum samples diluted 1 : 100 in 0.1 M PBS Tween containing 2% BSA were added to duplicate wells and incubated for 1 hour at room temperature.

Plates were washed again and then polyclonal and monoclonal antibodies diluted at an optimal dilution of 1 : 500 were added to duplicate antigen-coated wells; plates were incubated for an additional 1 hour at room temperature. The plates were then washed five times with TBS-Tween buffer. The enzyme reaction was started by adding 100 *μ*L of paranitrophenylphosphate in 0.1 mL diethanolamine buffer 1 mg/mL containing 1 mM MgCl_2_ and sodium aside at a pH of 9.8. The reaction was stopped 45 minutes later with 50 *μ*L of 1 N NaOH and the samples were read by an ELISA reader, and the optical densities (OD) were recorded.

For the determination of specificity of monoclonal and affinity-purified polyclonal antibodies in reaction with various food antigens, four wells of each 96-well plate were coated with 2% BSA alone but not the food antigens. After the addition of all other antigens, the mean OD of these wells were subtracted from all other reactions.

### 2.6. Determination of Immune Reactivity Scale

Two hundred and four proteins (see Table 1) were tested for seven target tissue antibodies in duplicate, leading to 2,856 antigen-antibody OD measurements. The results of each duplicate OD were averaged together for one OD value, and the CD of control wells, which was less than 0.15, was subtracted from all other measurements. Of the 2,856 OD measurements, the mean OD was 0.33 with a standard deviation (SD) of 0.53. The OD of 0.87 represented two standard deviations from the mean, the OD of 1.41 represented three standard deviations from the mean, and an OD of 1.95 represented four standard deviations from the mean. OD values below two standard deviations or less than 0.53 were labeled nonsignificant. OD values above two standard deviations but below three standard deviations at OD values of 0.66–0.86 were categorized as 1+ reaction. OD values above three standard deviations but less than four standard deviations at OD values of 0.87–1.07 were categorized as 2+ reactions. OD values above four standard deviations with OD values greater than 1.08 were categorized as 3+ reactions (see Table 2).

### 2.7. Binding of Serially Diluted Thyroid Antibody to Fixed Concentrations of Various Food Antigens

For the demonstration of anti-thyroid antibodies binding to food antigens, different wells of microtiter plates were first coated with optimal concentrations (10 *μ*g/well) of cashew, roasted cashew, latex hevein, egg yolk cooked, and seaweed. After the completion of all steps necessary for coating the plates, each monoclonal antibody in dilutions of 1 : 500–1 : 512,000 was added to two different rows of microtiter plates, and after completion of all ELISA steps, the optical densities (ODs) were measured.

### 2.8. Binding of Fixed Concentration of Antibody to Serially Diluted Food Antigens

Different food antigens in a concentration of 400 *μ*g/mL were serially diluted. Microtiter plates were coated with 0.03–40 *μ*g of each antigen in duplicate rows. After the incubation and blocking steps, the addition of a 1 : 500 dilution of monoclonal anti-thyroid antibody, and the completion of all ELISA steps, the OD were recorded.

### 2.9. Inhibition of Anti-Thyroid Antibody Binding to Food Antigen-Coated Plates by Different Concentrations of Food Antigens

Four different rows of three different microtiter plates were coated in this pattern: wells A1, B1, C1, and D1 were coated with T_3_; the other eleven wells of each row (A2–A12, B2–B12, C2–C12, and D2–D12) were coated with roasted cashew, egg, seaweed, or latex hevein. Controls and inhibitors were then added as follows: 100 *μ*L of serum diluent to wells A1-A2, B1-B2, C1-C2, and D1-D2; 100 *μ*L of diluent containing 100 *μ*g of T_3_ to wells A3, B3, C3, and D3; and 0.5–120 *μ*g of cashew, egg, seaweed, and latex hevein to, respectively, A4–A12, B4–B12, C4–C12, and D4–D12.

Plates were incubated at 37°C for 1 hour, and 100 *μ*L of mouse monoclonal anti-T_3_ was added to all 48 wells. After repeated incubation, washing, the addition of the secondary antibody, and completion of all the ELISA steps, the optical densities were recorded. Plate #2 was used for the addition of T_4_ and anti-T_4_ antibody. Plate #3 was used for the addition of Tg and anti-Tg with proper controls.

## 3. Results

### 3.1. Immune Reactivity between Affinity-Purified Polyclonal Antibodies and Food Antigens

The affinity-purified polyclonal antibody made against TSH-R and monoclonal antibody made against TPO did not react with any of 204 food proteins and TBG. But polyclonal antibody made against DIO2 reacted only with buckwheat with a 1+ reaction.

### 3.2. Reaction of Monoclonal Antibodies Made against Thyroglobulin, T_3_, and T_4_ with 204 Food Proteins

Using this monoclonal antibody against Tg resulted in a 3+ immune reaction with latex hevein (see Table 2).

Using monoclonal antibody against T_4_, our study found a significant list of food proteins that directly demonstrated immune reactivity with thyroxine. These foods include avocado, lemon and lime, cooked Brussels sprouts, seaweed, cooked zucchini, roasted and raw almond, cooked black bean, raw and roasted Brazil nut, cashew, roasted cashew, cashew vicilin, raw and roasted hazelnut, raw and roasted macadamia nut, mustard seed, roasted peanut, peanut butter, raw and roasted pistachio, gluten-free soy sauce, tofu, gelatin, cooked egg yolk, raw salmon, cooked tilapia, raw tuna, cooked tuna, cooked clam, cooked scallops, cooked squid (calamari), cooked shrimp, amaranth, and oats (see Table 2).

Similarly, using monoclonal antibody made against T_3_, we found a significant list of food proteins that directly demonstrated immune reactivity with triiodothyronine. These foods include avocado, latex hevein, lemon and lime, orange juice (pasteurized and concentrate), cooked Brussels sprouts, baked white potato, seaweed, radish, roasted almond, raw and roasted Brazil nut, cashew, roasted cashew, cashew vicilin, raw and roasted macadamia nut, mustard seeds, roasted peanut, peanut butter, raw and roasted pistachio, soy bean agglutinin, gluten-free soy sauce, tofu, roasted sunflower seeds, gelatin, cooked egg yolk, raw salmon, cooked tilapia, raw tuna, cooked tuna, cooked clam, cooked scallops, cooked squid (calamari), cooked shrimp, cow's milk, casein (alpha and beta), sesame, hemp, rye, barley, kamut, buckwheat, sorghum, millet, spelt, amaranth, quinoa, yeast, oats, corn, and rice (see Table 2).

### 3.3. Simultaneous Reactivity of Monoclonal Antibodies against Thyroid Target Sites

We selected 10 foods from Table 1 to see if the same immune reactivity with a specific food, for instance latex hevein, elicited by a monoclonal antibody made against T_3_, for example, would occur with a different monoclonal antibody. The data clearly shows that each monoclonal antibody shows different patterns of immune reactivity to the food antigens (see Table 3). In fact, TPO and TBG had no reactions to any food at all.

### 3.4. Demonstration of the Specificity of Anti-Thyroid Antibodies Binding to Different Food Antigens

**Figure 1:**
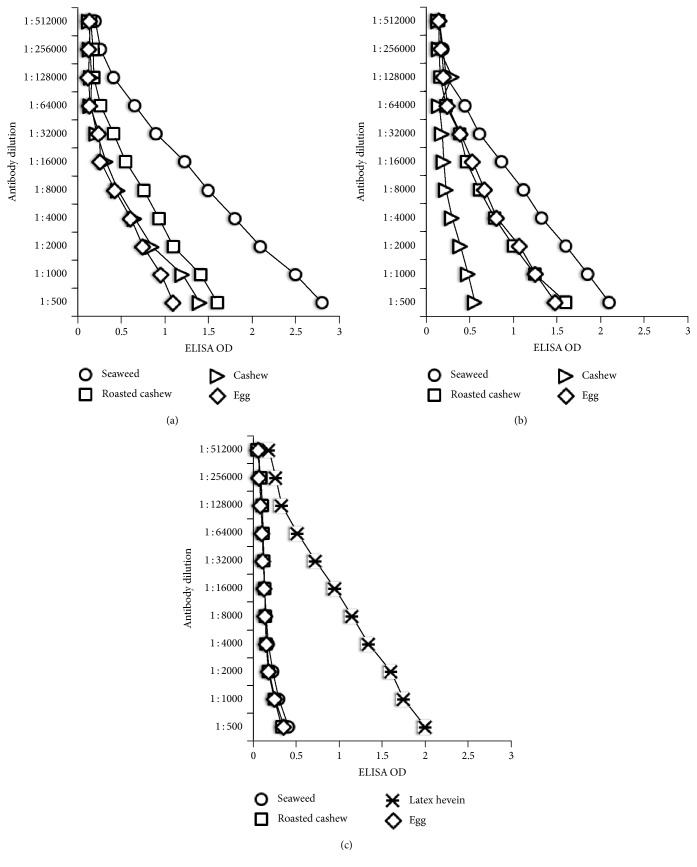
The binding of serially diluted T3 (a), T4 (b), and Tg (c) monoclonal antibodies to the same concentration of different food antigens.

**Figure 2:**
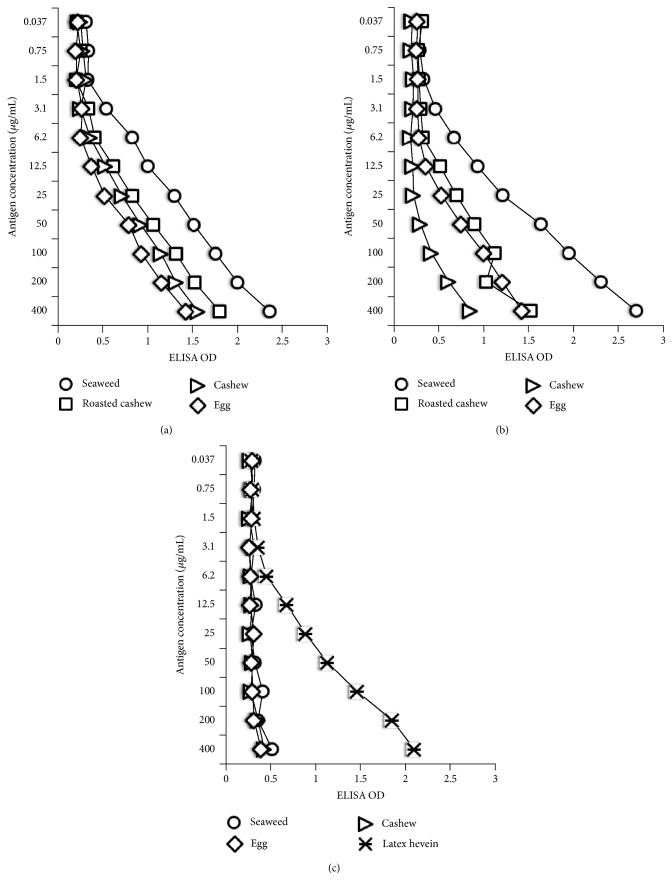
The binding of monoclonal antibody against T3 (a), T4 (b), and Tg (c) to serially diluted food antigens.

**Figure 3:**
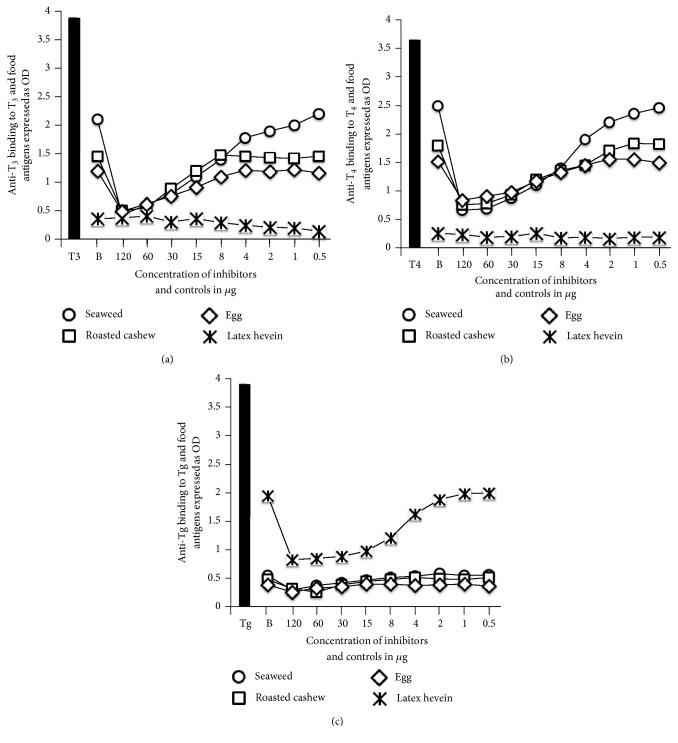
Inhibition of T3 (a), T4 (b), and Tg (c) antibodies with different concentrations of food antigens.

The specificity of these monoclonal anti-T_3_, anti-T_4_, and anti-Tg antibodies in binding to various food antigens was confirmed by dilution and inhibition studies. As shown in Figures 1(a), 1(b), and 1(c), in proportion to the dilutions of the monoclonal antibodies, the OD or immune reactions to the food antigens decline significantly. For example, the reaction of anti-T_3_ antibody at a dilution of 1 : 500 with seaweed gives an OD of 2.8, a dilution of 1 : 16,000 gives an OD of 1.23, and a dilution of 1 : 512,000 gives an OD of 0.2, which is equivalent to the background of the ELISA (see Figure 1(a)). Similar results were obtained with the reaction of diluted T_4_ with various food antigens (see Figure 1(b)). Interestingly, using anti-T_4_ antibody, we found significant differences between the immune reactivity of raw cashew and roasted cashew (Figure 1(b)). The binding of anti-Tg antibody to latex hevein, but not to seaweed, egg, or cashew, and the proportion of the binding to antibody dilution are shown in Figure 1(c). Furthermore, when fixed amounts of antibodies made against T_3_, T_4_, and Tg were added to plates coated with food concentrations of 0.037–400 *μ*g/mL, the ELISA OD increased in proportion to the concentration of the food antigens (see Figures 2(a), 2(b), and 2(c)). The reaction of anti-T_4_ with serially diluted roasted cashew was stronger than with raw cashew (see Figure 2(b)). To further demonstrate the specificity of these antigen-antibody reactions, different amounts of food antigens (inhibitors) in concentrations of 0.5–120 *μ*g or controls were added in the liquid phase of plates that contained the optimal concentrations of food antigens in solid phase. The addition of anti-T3, anti-T4, or anti-Tg antibodies to the mixture resulted in a significant inhibition of thyroid antibody binding to some food antigens on the plates. This inhibition of antigen-antibody reaction was more obvious when higher concentrations of food antigens were used in the liquid phase (Figures 3(a), 3(b), and 3(c)).

## 4. Discussion

Our laboratory study found dietary proteins that share amino acid sequence homology and have the potential to play a role in cross-reactivity with thyroid target sites. This may serve to fill a knowledge gap and may have identified a starting place for further research into the relationship between immunological responses to dietary proteins and autoimmune thyroid disease. We identified immune reactivity between antibodies made against various thyroid target antigens and food proteins and target sites on various locations of the thyroid axis. This potential antibody against specific thyroid axis sites with food antigens can lead to the possibilities that some dietary proteins may play a role in autoimmune thyroid disease. Our study identified T_4_, T_3_, Tg, and 5-deiodinase antibodies that reacted with purified food antigens. These reactions suggest immunological cross-reactivity between food immune reactions and thyroid axis sites.

Interestingly, although many pathogenic organisms have shown direct cross-reactivity with both TSH-R and TPO, our study found that none of the 204 foods tested demonstrated any immune reactive response to these key autoimmune target sites associated with Graves' and Hashimoto's disease, with the exception of latex hevein for thyroglobulin. Hevein is a lectin-like protein derived from* Hevea brasiliensis *(rubber tree). Although this lectin-like protein is not found in food, it is associated with the latex-fruit syndrome. Approximately 30–35% of individuals who are allergic to natural rubber latex show cross-reactive IgE hypersensitivity to plant sources such as avocado, chestnut, bell pepper, kiwi, peach, and tomato. Immune cross-reactivity with latex or latex-fruit syndrome may potentially lead to Tg cross-reactivity in susceptible individuals who are reactive to natural rubber latex and associated foods [28].

Although direct food reactions did not occur with TSH-R and TPO most commonly associated with Graves' disease and Hashimoto's, there was significant cross-reactivity between both T_4_ and T_3_ hormones found within the thyroid gland with 25–35 food antigens (Table 2) [29, 30].

We also did not identify immune reactions to TBG in our study, suggesting food immune reactivity does not appear to have a role in thyroid hormone transport dysregulation or on ratios of thyroid hormones bound to protein and, therefore, foods should not impact ratios of bound compared to unbound thyroid hormones or biomarkers such as T_3_ uptake.

One reaction was identified between Type II 5-deiodinase (DIO_2_), thereby suggesting that food immune reactivity with buckwheat may potentially impact T_4_ to T_3_ thyroid conversion associated with nonthyroidal illness syndrome (NTI), or euthyroid sick syndrome. These findings suggest a potential new mechanism for NTI not reported in the literature to the best of our knowledge.

Most of the cross-reactive reactions occurred with T_4_ and T_3_, and many of the cross-reactive foods overlapped with both hormones, as T_4_ and T_3_ have very similar amino acid homology, especially involving iodine sequencing [31]. Some common patterns were identified with these reactions, including cross-reactions to many gluten-containing foods and grains and foods that contain iodine structural sequences similar to thyroid hormones, such as seaweed, white potato, nuts (almond, cashew, hazelnut, and peanut), seafood, and more (Table 2).

There was also overlap between T_4_ and T_3_ foods not associated with iodine, such as cooked Brussels sprouts, lemon and lime, radish, tofu, gluten-free soy sauce, and cooked zucchini. Some of these food proteins have been classified as goitrogens; however, their impact on thyroid function may be due to immune cross-reactivity instead of interference with iodine uptake, as proposed in the goitrogenic model. This is especially probable given that some of these goitrogenic foods were tested in a modified cooked state to remove goitrogenic properties such as glucosinolates. Immunological cross-reactivity with foods listed as goitrogenic may explain some of the observed adverse reactions that occur in small subsets of thyroid patients, although the mechanisms now appear to be immunological rather than goitrogenic.

Additionally, there were many cross-reactive foods with T_4_ and T_3_ that did not overlap with each other. Although T_3_ and T_4_ have some structural similarity with each other, T_4_ has two independent conformations in the crystal lattice with significant differences in the outer phenyl ring geometry when compared to T_3_. The major differences between T_4_ and T_3_ structures are shortened C4′-O4′ bond contraction of the C3′-C4′-C5′ angle and an increase in the C3′ and carbon C5′ angles of T_4_ [31]. These differences may explain why certain food proteins only have cross-reactive reactions with either T_3_ or T_4_. Overall, this simultaneous reaction of three different monoclonal antibodies made against Tg, T_3_, and T_4_ with some food antigens but not others is the best indication for the specificity of the immune reactions between antibodies made against thyroid target antigens and different food proteins (see Tables 2 and 4).

The specificity of these anti-thyroid antibodies binding to antigenic determinants of food antigens was shown by serial dilution of food antigens or different concentrations of monoclonal antibody with the same concentration of food antigens in the solid phase. The dose response curves shown in Figures 1(a), 1(b), 2(a), and 2(b), with seaweed first followed by roasted cashew, and cooked egg yolk, and the curves in Figure 1(c) that show a strong response from latex hevein are supportive of the specificity of these antigen-antibody reactions. To further confirm this reaction of antibodies with food antigens, inhibition studies were conducted with different concentrations of food antigens in the liquid phase and the addition of monoclonal thyroid antibodies. As shown in Figures 3(a) and 3(b), the addition of 120 *μ*g of seaweed to the liquid phase resulted in 76% inhibition in the binding of anti-T_3_ and 80% in the binding of anti-T4 to the food antigens (Figures 3(a) and 3(b)). The inhibition of Tg antibody to seaweed was not significant since, to begin with, the anti-Tg antibody did not react to seaweed (see Figure 3(c)). In contrast, the inhibition of anti-Tg binding to latex hevein was very significant (>50%) when 15–120 *μ*g of antigen was added to the liquid phase. Overall, this inhibition of anti-thyroid antibody binding to different food antigens was reversed in proportion to the lower concentration of immune reactive foods in the liquid phase. The results of these experiments support the proposition that the binding of monoclonal antibodies to T3, T4, and Tg to different foods as shown in Tables 1–3 is specific, and hence cross-reactivity between thyroid tissue antigens and various food antigens should be taken seriously.

## 5. Conclusions

The results of our study identified immune reactivity between T_3_, T_4_, DIO_2_, thyroglobulin, and many food proteins. These immune reactions may explain the etiology of some cases of food interactions with both thyroid hormone replacement and thyroid hormone metabolism. Theoretically, these food protein reactions may contribute to autoimmune reactivity in a subset of autoimmune thyroid disease patients, a subject that warrants further research.

It is doubtful that the consumption of potentially reactive food proteins alone would induce an inflammatory response on thyroid axis target sites. The consumption of dietary proteins in combination with other factors such as digestive enzymes is necessary to induce immunological reactions. First, dietary proteins cannot have an impact on antigenic-antibody models if properly digested, unless the individual is immunologically reactive to those foods and thereby producing antibodies to those specific dietary proteins due to a failure in oral tolerance to those specific undigested food antigens [32]. If the individual has oral tolerance to ingested proteins and does not produce significant antibodies to those proteins, then no reaction would be expected. Second, antigen-antibody reactions alone are not solely responsible for pathogenic reactions. Cellular immunity, immunological tolerance, human leukocyte antigen (HLA) allele, and other factors are involved with pathogenic immune reactions to dietary proteins. Therefore, antibodies binding to antigens are not always pathogenic; however, the ability of specific monoclonal and polyclonal antibodies to bind with purified proteins suggests the potential for immunological cross-reactivity in a subset of susceptible individuals.

Further research should be conducted to evaluate the specific epitope for each of these foods and thyroid axis target sites. Additionally, immunological factors such as tolerance, T-cell polarization, and other factors that may induce susceptibility to dietary protein immune reactivity need to be investigated. Individual case studies and clinical trials will be required to assess whether any actual clinical role exists for these food proteins in thyroid-associated reactions. The results of our research provide a list of susceptible dietary proteins that may immunologically impact thyroid interactions and warrant further study. These results provide a first step in narrowing down a list of specific dietary proteins that, due to protein cross-reactivity, may potentially have an impact on autoimmune thyroid disease.

## Figures and Tables

**Table 1:** Dietary proteins screened for immune reaction.

*DAIRY and eggs, modified*
Alpha-casein and beta-casein
Cow's milk
Chocolate milk
Egg White, boiled
Egg Yolk, boiled
Goat's milk
Milk butyrophilin
Soft cheese + hard cheese
Whey protein
Yogurt

*GRAINS, raw and modified*
Amaranth
Buckwheat
Casomorphin
Oats
Quinoa
Rice
Rice, white + brown, boiled
Rice cake
Rice protein
Rice endochitinase
Rye, Barley, Spelt, Polish Wheat
Sesame
Sorghum
Tapioca
Teff
Wild rice, boiled
Wheat + alpha-gliadins
Yeast miller
Hemp

*BEANS, modified*
Black bean, boiled
Bean agglutinins
Dark chocolate + cocoa
Fava bean, boiled
Garbanzo bean, boiled
Kidney bean, boiled
Lentil, boiled
Lentil lectin
Lima bean, boiled
Pinto bean, boiled
Soybean agglutinin
Soybean oleosin + Aquaporin
Soy sauce, gluten-free
Tofu

*Nuts and seeds, raw and modified*
Almond
Almond, roasted
Brazil nut, raw + roasted
Cashew
Cashew, roasted
Cashew vicilin
Chia seed
Flax seed
Hazelnut, raw + roasted
Macadamia nut, raw + roasted
Mustard seed
Pecan, raw + roasted
Peanut, roasted
Peanut butter
Peanut agglutinin
Peanut oleosin
Pistachio, raw + roasted
Pumpkin seeds, roasted
Sesame albumin
Sesame oleosin
Sunflower seeds, roasted
Walnut

*Vegetables, raw and modified*
Artichoke, boiled
Asparagus
Asparagus, boiled
Beet, boiled
Bell Pepper
Broccoli
Broccoli, boiled
Brussels sprouts, boiled
Cabbage, red + green
Cabbage, boiled
Canola oleosin
Carrot
Carrot, boiled
Cauliflower, boiled
Celery
Chili pepper
Corn + aquaporin, boiled
Popped corn
Corn oleosin
Cucumber, pickled
Eggplant, boiled
Garlic
Garlic, boiled
Green bean, boiled
Lettuce
Mushroom, raw + boiled
Okra, boiled
Olive, green + black, pickled
Onion + scallion
Onion + scallion, boiled
Pea, boiled
Pea Protein
Pea Lectin
Potato, white, baked
Potato, white, fried
Pumpkin + squash, boiled
Radish
Safflower + sunflower Oleosin
Seaweed
Spinach + aquaporin
Tomato + aquaporin
Tomato paste
Yam + sweet potato, baked
Zucchini, boiled

*Fruit, raw and modified*
Apple
Apple cider
Apricot
Avocado
Banana
Banana, boiled
Latex hevein
Blueberry
Cantaloupe + honeydew melon
Cherry
Coconut, meat + water
Cranberry
Date
Fig
Grape, red + green
Red wine
White wine
Grapefruit
Kiwi
Lemon + lime
Mango
Orange
Orange juice
Papaya
Peach + nectarine
Pear
Pineapple
Pineapple bromelain
Plum
Pomegranate
Strawberry
Watermelon

*Fish and seafood, raw and modified*
Cod, baked
Halibut, baked
Mackerel, baked
Red Snapper, baked
Salmon
Salmon, baked
Sardine + anchovy, cooked
Sea Bass, seared
Tilapia, baked
Trout, baked
Tuna
Tuna, seared
Whitefish, baked
Crab + lobster, boiled
Imitation crab
Clam, boiled
Oyster, boiled
Scallops, seared
Squid (calamari), seared
Shrimp, seared
Shrimp tropomyosin
Parvalbumin

*Meat, modified*
Beef, boiled medium
Chicken, boiled
Lamb, baked
Pork, baked
Turkey, baked
Gelatin
Meat glue

*Herbs, raw*
Basil
Cilantro
Cumin
Dill
Ginger
Oregano
Parsley
Rosemary
Thyme

*Spices, raw*
Cinnamon
Clove
Mint
Nutmeg
Paprika
Turmeric (curcumin)
Vanilla

*GUMS*
Carrageenan
Gum guar
Gum tragacanth
Locust bean gum
Mastic gum + gum Arabic
Xanthan gum

*Brewed beverages and additives*
Coffee bean protein, brewed
Instant coffee
Black tea, brewed
Green tea, brewed
Honey, raw + processed
Beta-glucan
Food coloring

**Table 2:** Immunoreactivity of monoclonal antibodies to T3 and T4 with food proteins.

Food protein	T_3_	T_4_
Almond	−	++
Almond roasted	++	+++
Amaranth	+++	+
Avocado	+	+
Barley	++	−
Black bean cooked	−	+
Brazil nut raw & roasted	+	+++
Brussels sprouts cooked	+	++
Buckwheat	+++	−
Casein (*α* & *β*)	+	−
Cashew	++	+
Cashew roasted	++	+++
Cashew vicilin	+	+++
Chocolate	++	−
Clam cooked	+	+++
Coffee	+++	−
Corn	+++	−
Cow's milk	++	−
Egg Yolk cooked	++	+++
Gelatin	++	+++
Hazelnut raw and roasted	−	+
Hemp	+++	−
Kamut	+++	−
Latex hevein	+++	−
Lemon and lime	+	+
Macadamia nut raw and roasted	++	++
Millet	++	−
Mustard seed	+++	+++
Oats	+++	++
Orange Juice pasteurized or concentrate	+++	−
Peanut butter	++	++
Peanut roasted	++	+
Pistachio raw and roasted	+	++
Potato	+++	−
Potato white baked	+	−
Quinoa	+++	−
Radish	+	−
Rice	+++	−
Rye	++	−
Salmon raw	+	+++
Scallops cooked	+++	+++
Seaweed	+++	+++
Sesame	+	−
Shrimp cooked	++	+++
Sorghum	+++	−
Soy Bean agglutinin	+	−
Soy sauce gluten-free	+	+++
Spelt	++	
Squid (calamari) cooked	+++	+++
Sunflower seeds roasted	+	−
Tapioca	+++	−
Tofu	+++	+++
Tilapia cooked	+	+++
Tuna cooked	+	+++
Tuna raw	++	+++
Yeast	+	−
Zucchini cooked	−	++

+ = 0.66–0.86; ++ = 0.87–0.107; +++ ≥ 1.08.

**Table 3:** Example of 10 selected foods and their degrees of reactivity with monoclonal antibodies against Tg, T3 and T4.

	Tg	T_3_	T_4_
Latex hevein	+++	+++	−
Kamut	−	+++	−
Soy sauce	−	+	+++
Gelatin	−	++	+++
Scallops	−	+++	+++
Cashew, roasted	−	++	+++
Cashew, vicilin	−	+	+++
Coffee protein	−	+++	−
Brazil nut	−	+	+++
Almond	−	−	++

**Table 4:** Reaction of monoclonal and polyclonal antibodies against 204 different foods.

Antibodies	Degree of reaction			
	−	+	++	+++
Polyclonal antibody against TSH-R	204	0	0	0
Polyclonal antibody against DIO_2_	203	1	0	0
Monoclonal antibody against TPO	204	0	0	0
Polyclonal antibody against TBG	204	0	0	0
Polyclonal antibody against Tg	203	0	0	1
Polyclonal antibody against T3	151	18	16	19
Polyclonal antibody against T4	172	7	7	18
